# MoodHacker Mobile Web App With Email for Adults to Self-Manage Mild-to-Moderate Depression: Randomized Controlled Trial

**DOI:** 10.2196/mhealth.4231

**Published:** 2016-01-26

**Authors:** Amelia J Birney, Rebecca Gunn, Jeremy K Russell, Dennis V Ary

**Affiliations:** ^1^ ORCAS Eugene, OR United States

**Keywords:** depression, cognitive behavioral therapy, behavioral activation, positive psychology, mobile apps, Internet, computers

## Abstract

**Background:**

Worldwide, depression is rated as the fourth leading cause of disease burden and is projected to be the second leading cause of disability by 2020. Annual depression-related costs in the United States are estimated at US $210.5 billion, with employers bearing over 50% of these costs in productivity loss, absenteeism, and disability. Because most adults with depression never receive treatment, there is a need to develop effective interventions that can be more widely disseminated through new channels, such as employee assistance programs (EAPs), and directly to individuals who will not seek face-to-face care.

**Objective:**

This study evaluated a self-guided intervention, using the MoodHacker mobile Web app to activate the use of cognitive behavioral therapy (CBT) skills in working adults with mild-to-moderate depression. It was hypothesized that MoodHacker users would experience reduced depression symptoms and negative cognitions, and increased behavioral activation, knowledge of depression, and functioning in the workplace.

**Methods:**

A parallel two-group randomized controlled trial was conducted with 300 employed adults exhibiting mild-to-moderate depression. Participants were recruited from August 2012 through April 2013 in partnership with an EAP and with outreach through a variety of additional non-EAP organizations. Participants were blocked on race/ethnicity and then randomly assigned within each block to receive, without clinical support, either the MoodHacker intervention (n=150) or alternative care consisting of links to vetted websites on depression (n=150). Participants in both groups completed online self-assessment surveys at baseline, 6 weeks after baseline, and 10 weeks after baseline. Surveys assessed (1) depression symptoms, (2) behavioral activation, (3) negative thoughts, (4) worksite outcomes, (5) depression knowledge, and (6) user satisfaction and usability. After randomization, all interactions with subjects were automated with the exception of safety-related follow-up calls to subjects reporting current suicidal ideation and/or severe depression symptoms.

**Results:**

At 6-week follow-up, significant effects were found on depression, behavioral activation, negative thoughts, knowledge, work productivity, work absence, and workplace distress. MoodHacker yielded significant effects on depression symptoms, work productivity, work absence, and workplace distress for those who reported access to an EAP, but no significant effects on these outcome measures for those without EAP access. Participants in the treatment arm used the MoodHacker app an average of 16.0 times (SD 13.3), totaling an average of 1.3 hours (SD 1.3) of use between pretest and 6-week follow-up. Significant effects on work absence in those with EAP access persisted at 10-week follow-up.

**Conclusions:**

This randomized effectiveness trial found that the MoodHacker app produced significant effects on depression symptoms (partial eta^2^ = .021) among employed adults at 6-week follow-up when compared to subjects with access to relevant depression Internet sites. The app had stronger effects for individuals with access to an EAP (partial eta^2^ = .093). For all users, the MoodHacker program also yielded greater improvement on work absence, as well as the mediating factors of behavioral activation, negative thoughts, and knowledge of depression self-care. Significant effects were maintained at 10-week follow-up for work absence. General attenuation of effects at 10-week follow-up underscores the importance of extending program contacts to maintain user engagement. This study suggests that light-touch, CBT-based mobile interventions like MoodHacker may be appropriate for implementation within EAPs and similar environments. In addition, it seems likely that supporting MoodHacker users with guidance from counselors may improve effectiveness for those who seek in-person support.

**Trial Registration:**

ClinicalTrials.gov NCT02335554; https://clinicaltrials.gov/ct2/show/NCT02335554 (Archived by WebCite at http://www.webcitation.org/6dGXKWjWE)

## Introduction

### Background

Major depressive disorder is one of the most prevalent mental conditions to afflict adults in the United States, with estimates in the United States for major depression of 16.6% for lifetime occurrence and 6.7% for a 1-year period [1,2]. Worldwide, depression is rated as the fourth leading cause of disease burden, and the World Health Organization projects that by 2020 depression will rank as the second leading cause of disability [3,4]. The prevalence of mild-to-moderate or subclinical depression is equal to, or greater than, major depressive disorder, with lifetime rates up to 26% and annual prevalence of 5-10% [5,6]. Subclinical depression is associated with substantial functional impairment, including poor work performance [3,7-9]. Further, subclinical depression is associated with a two- to five-fold increased risk of full-syndrome depressive disorders [10-13].

Based on data from 2010, depression-related costs in the United States exceeded US $210.5 billion, with employers incurring US $102 billion in losses due to presenteeism (US $78.7 billion), absenteeism (US $23.3 billion), and disability, and another US $98.9 billion incurred as direct medical costs [14]. Each year, US employers lose approximately 32 workdays per depressed employee to presenteeism [14]. Approximately 40% of direct medical costs are due to major depressive disorder, 10-11% are due to other depressive conditions, and roughly half of costs (48-51%) are due to comorbid physical or psychiatric conditions, such as pain and sleep disorders [14]. In addition, the economic costs of subclinical depression are considerable, approximately two-thirds the per capita costs of major depression [15-18].

Because most adults who suffer from depression never receive treatment [19], there is a need to develop interventions that can be more widely disseminated, such as through channels like employee assistance programs (EAPs) and directly to individuals who will not seek face-to-face care. Interventions that reduce the performance-impairing symptoms of subclinical depression and prevent the onset of major depression can improve employee well-being, while reducing health care costs and improving productivity [20]. EAPs offer services specifically designed to improve and/or maintain workplace productivity, including individual mental and behavioral health services offered to employees and family members experiencing personal difficulties, like depression. With wide reach into medium-to-large US employers and rapidly growing reach worldwide, EAPs offer a meaningful channel for delivering effective interventions as part of a larger population health management strategy.

### Cognitive Behavioral Therapy for Depression

The Coping with Depression (CWD) cognitive behavioral therapy (CBT) skills-training program [21,22] is based on behavioral [23,24] as well as cognitive formulations of depression [25-27]. The CWD skills-training program combines cognitive and behavioral strategies aimed at ameliorating problems common to depressed individuals (eg, pessimism; internal, global, and stable attributions for failure; low self-esteem; low engagement in pleasant activities; poor social skills; anxiety and tension; low social support; and increased conflict) with a focus on awareness of specific and current actions and cognitions as targets for change. CWD-based interventions are based on the premise that activating a variety of coping skills and strategies allows depressed individuals to effectively address the diverse personal and environmental triggers that underlie their depressive symptoms.

The CWD approach has been validated for use with a variety of age, gender, and race/ethnic groups and using a variety of delivery methods, including as a guided self-help intervention [28]. Based on a meta-analysis of 18 CWD-based intervention studies, the approach has been associated with clinically significant effects ranging from *d*=0.28 to 0.62, depending on the outcome measure used [28]. In trials that targeted adults with subthreshold depression, CWD participants were 38% less likely to escalate to full-syndrome depression [28].

### Positive Psychology Interventions for Depression

Cognitive and behavioral approaches to mood have evolved from a focus on treating disorders to including an emphasis on promoting positive emotion and experiences. Positive psychology interventions (PPIs), such as mindful self-awareness, gratitude expression, and identifying and utilizing strengths, have been shown to impact both well-being and depressive symptoms [29-31]. Mindfulness and acceptance are increasingly integrated in CBT approaches to depression self-management, drawing attention to both increasing positive cognitions and experiences, and accepting or releasing negative cognitions. Mindfulness-based interventions are particularly effective for managing stressors and enhancing positive emotions, resulting in psychological and social benefits and reducing depression symptoms [31-35]. PPIs that focus on building conscious awareness and expression of gratitude increase positive emotion and yield a wide variety of physical and affective benefits [31,36-38]. Identifying and using one’s strengths has been shown to have robust emotional effects, and setting strength-based goals and plans has been shown to increase optimism and reduce depression, with long-lasting effects [31,39-41].

### Web and Mobile Delivery of Depression Interventions: Efficacy

Internet-delivered CBT-based programs for depression symptom management have been shown to be effective both for adults with major depressive disorder and those with elevated, but subthreshold, depression symptoms, although one recent review identified concerns with dropout and lack of long-term effects [42-45]. Traditional computer- and Internet-based CBT programs typically involve four to 12 lessons or modules delivered in sequence, either weekly or self-paced [43,46]. Effect sizes have been estimated at *d*=0.44-1.90 for clinician-supported, and *d*=0.21-0.70 for self-guided, interventions [42-45,47,48]. One review reports somewhat larger effects in studies targeting populations with clinically significant depression symptoms (*d*=0.42-0.65) than for populations with mild-to-moderate symptoms (*d*=0.30-0.53) [43].

To date, very few randomized clinical trials of mobile apps targeting depression have been published in the peer-reviewed literature [49]. We found only one such study specifically targeting adults with mild-to-moderate depression [50,51]. In this study, participants who used the CBT-based myCompass app, delivered via mobile phone and Internet, reported improvements in depression and anxiety at 7-week post-test compared to an attention control intervention and a waiting list condition. Treatment gains were maintained at 3-month follow-up, and improvements in the attention control condition matched those of the myCompass group [50].

The SuperBetter mobile Web app was evaluated in a three-arm randomized controlled trial, with results showing the original, “general” version of SuperBetter—with activities focused on self-esteem and social support—more effective at reducing depressive symptoms over both a combined SuperBetter-plus-CBT and positive psychology strategies condition and the waiting list condition. Ly et al [52] found no between-group effects between mobile-based behavioral activation and mindfulness apps for depression. Kauer et al [53] and Reid et al [54] found no differences in the Depression, Anxiety, and Stress Scale scores between a mobile app with ecological momentary assessment (EMA)-based emotional self-awareness training and feedback and an attention control condition. Watts et al [55] reported statistically significant large within-group effect sizes for depression symptoms in a nonrandomized study of their CBT-based Get Happy mobile app, which was derived from, and evaluated against, its Web-based predecessor, The Sadness Program. Burns et al [56] conducted a single-group pre/post pilot of Mobilyze!, a website with a mobile EMA component, finding large within-group effect sizes on depressive symptomatology.

The CWD-based Overcoming Depression on the Internet program provided early evidence that a Web-based program with reminders was effective in reducing depression symptoms, particularly among individuals with mild-to-moderate depression [57,58]. Spek et al [59] found similar improvements among individuals over 50 years old with subthreshold depressive symptoms from a CWD-based, self-administered Internet treatment program and those receiving CWD-based group therapy, both significantly greater than the improvement seen in waiting list controls. Treatment effects for the Internet-based group were maintained 1 year following treatment [60].

### Web and Mobile Delivery of Depression Interventions: Access

Internet delivery of mental health interventions offers individuals with online technology broad access to evidence-based treatment. Mobile apps offer an additional channel to increase access further, both as self-guided tools and as those supported by counseling professionals, such as EAPs. Globally, use of mobile and broadband mobile services is rising, while fixed broadband service is slowing, especially in developing countries [61]. Worldwide penetration of broadband mobile services doubled between 2011 and 2014 to an estimated 32% (84% in developed countries and 21% in developing countries) [61]. In 2014, there were nearly 7 billion active mobile subscriptions worldwide, with 2.3 billion using broadband services [61]. In 2015, 64% of Americans owned a mobile phone and 19% used it for their only or primary access to the Internet [62]. Mobile phone-dependent Americans tend to be younger, nonwhite, and have low income and education [62]. Capitalizing on mobile Internet access as a delivery channel for evidence-based mental health interventions is critical.

Mobile interventions offer the potential for anytime, anywhere convenience and the ability to promote regular use of behavioral and cognitive self-management strategies known to impact mood. Consumers express high interest in, and willingness to use, mobile phones and short message service (SMS) text messaging to monitor and manage symptoms and cite essential considerations such as ease of use, privacy, and security [63-66]. Proven mobile apps can provide a tool for clinical professionals to use with help-seeking clients, while also increasing access and retention over in-person mental health services [67,68]. Unfortunately, although the consumer app stores offer over 200 depression-specific apps, judging the credibility and efficacy of those apps is difficult without clinical validation [69].

### MoodHacker Mobile Intervention

The MoodHacker mobile intervention is one of few clinically validated CBT-based depression self-management mobile apps currently available. MoodHacker is designed to directly activate key cognitive and behavioral skills from the validated CWD program [21] and positive psychology strategies [70] (eg, mood and positive activity planning and tracking, cognitive restructuring, mindful self-awareness, gratitude expression, and identifying and utilizing strengths). In contrast to the more common lessons-based structure of many online and mobile CBT programs, MoodHacker is optimized for a brief daily interaction with the high-quality production value and mobile user experience common in consumer mobile apps.

### Objectives

To demonstrate the efficacy of this light-touch, mobile, Web CBT-based experience, we compared MoodHacker as a fully self-guided intervention to an alternative-treatment control group that received an email with links to vetted online information about depression. It was hypothesized that MoodHacker users would experience reduced depression symptoms and negative cognitions, and increased behavioral activation, knowledge of depression, and functioning in the workplace. This study of the MoodHacker intervention extends the evidence base for CBT-based mobile interventions for adults with mild-to-moderate depressive symptoms. Because the MoodHacker mobile Web app was designed primarily to target employees who present with depression through their EAP, the extent to which the program effects generalize to users with and without access to EAP support was an important research question.

## Methods

### Research Design

The efficacy of the MoodHacker mobile Web app intervention was assessed with a randomized controlled trial (ClinicalTrials.gov NCT02335554) with two factors: condition and EAP access (ie, subjects who had access to an EAP versus those who did not). See Multimedia Appendix 1 for the CONSORT-EHEALTH checklist for the trial [71]. There were three assessments: baseline, follow-up at 6 weeks after baseline, and follow-up at 10 weeks after baseline. After screening into the study, agreeing to the online informed consent, and submitting the baseline assessment, participants were blocked on race/ethnicity and randomized within block into either (1) treatment intervention group (n=150), which used the MoodHacker intervention for 6 weeks, or (2) alternative care group (n=150), which received links to six websites with information about depression. All study protocols, the consent process, and subject communications were reviewed and approved by the ORCAS Institutional Review Board (IRB) for protection of human subjects. There were no changes to the trial design after the trial commenced.

### Participants

**Figure 1:**
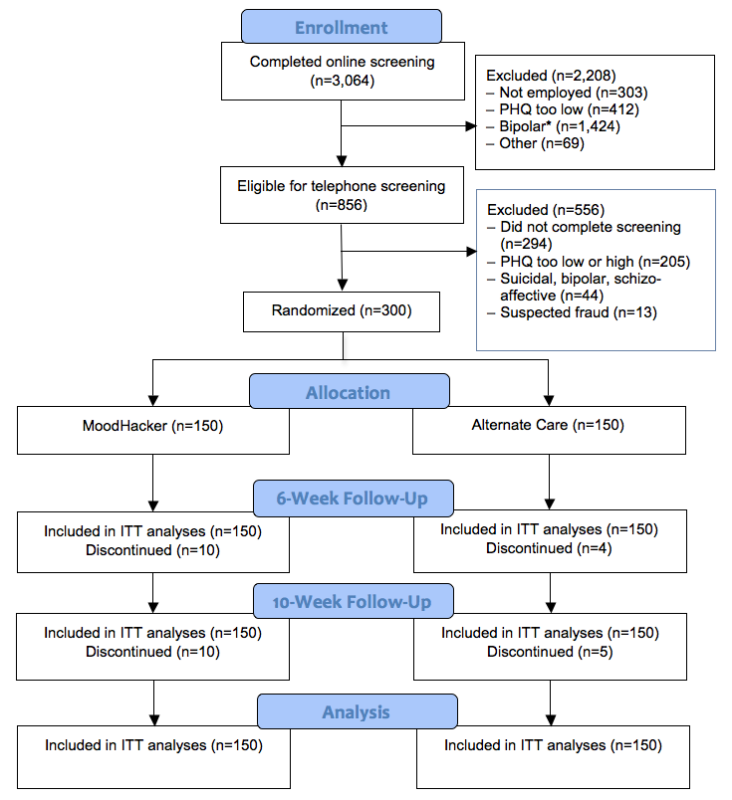
CONSORT diagram for the MoodHacker randomized trial.

Inclusion criteria for participation were defined as follows: (1) 18 years or older, (2) mild-to-moderate depressive symptoms as measured by the Patient Health Questionnaire-9 (PHQ-9) (score of 10-19), (3) not currently suicidal or meeting criteria for bipolar or schizoaffective disorder, (4) employed at least part time, (5) English speaking, and (6) have access to a high-speed Internet connection. Eligibility was assessed using a two-stage screening protocol. In total, 3064 individuals completed an online screening survey, which included questions on demographics, technology access, and depression symptoms (using the Patient Health Questionnaire-2 [PHQ-2]), as well as brief screening for bipolar and schizoaffective disorders. Of those, 856 (27.94%) qualified for secondary telephonic screening to more fully assess their depression symptoms with the PHQ-9 and to confirm their assessments for bipolar and schizoaffective disorders. Of those 856 who qualified, 294 (34.3%) individuals failed to complete the secondary screening, 205 (23.9%) did not meet the PHQ-9 criteria, 44 (5.1%) endorsed suicidal ideation or showed symptoms of bipolar or schizoaffective disorder, and 13 (1.5%) were dropped due to suspicion of fraud (see below). A total of 300 individuals out of 856 (35.0%) from 37 US states completed the online baseline assessment and were randomly assigned to one of two conditions: treatment or alternative care. See Figure 1 for a CONSORT diagram describing study enrollment and allocation.

### Study Setting and Data Collection

Participants were recruited from August 2012 through April 2013 in partnership with several organizations, including Chestnut Global Partners EAP as our primary recruitment partner. Non-EAP recruitment partners included Hope to Cope, Esperanza, Mental Health America, National Alliance on Mental Illness, LIVESTRONG, eHow, other eHealth websites, Chamber of Commerce offices, employee support organizations, and Craigslist. Outreach was conducted via the Chestnut EAP call center, print ads, online postings and ads, email listservs, and flyers. All interested potential participants were directed to an informational website that described the broad characteristics of the study’s purpose, activities, and compensation, concluding with an online screening survey.

All subjects participated fully online from the location(s) of their choice, using their own Internet-capable computers and mobile devices. All self-report, online screening and assessment data were collected via encrypted websites. Upon prequalification based on the initial screening survey, research staff conducted telephone interviews with potential participants to determine eligibility per the inclusion criteria referenced above. Potential participants who reported current suicidal ideation and/or bipolar or schizoaffective disorder during screening were offered appropriate resources according to an IRB-approved crisis protocol and were excluded from the study. The number of participants who self-identified with bipolar disorder symptoms in the initial screening survey was elevated due to suspected fraudulent individuals attempting to qualify with severe symptoms.

Throughout the study, individuals who reported current suicidal ideation and/or severe depression symptoms (PHQ-9 > 19) were contacted by telephone and offered appropriate resources according to an IRB-approved crisis protocol. Calls were made to 114 individuals (50 treatment, 64 control) and all remained in the study. No subjects reported suicide risk severe enough to transfer to a suicide hotline. Nor did any subjects report any adverse events related to the use of the MoodHacker app via email or during follow-up calls. Although research assistants were aware of group assignment, all other interactions with subjects were delivered by emails that were standardized across groups and fully automated to avoid differential interactions by group assignment. All other research team members were blinded and, aside from crisis calls, no research team members had direct interaction with subjects after randomization.

Lack of direct contact with study participants in a fully online study lends itself to potential participants self-reporting false information to qualify (eg, same name or IP address shows inconsistent age, gender, race, and/or ethnicity across multiple attempted screenings). To identify these individuals, demographic and contact data were cross-checked for fraudulent information against other individuals in the study database, as well as in our database of over 20,000 records of previous Internet study applicants. Those suspected of submitting fraudulent data were dropped from the study prior to randomization.

To enhance sample representativeness in each experimental condition, qualified participants were blocked on race/ethnicity and then randomly assigned within each race/ethnicity block to condition—treatment or alternative care—using the random number function in our subject database. Emails indicating group assignment and linking participants to the online informed consent form were auto-generated in the database and sent to participants by a research assistant. Upon completion of the consent form, they were immediately linked to the online baseline self-assessment. Participants also completed online follow-up self-assessments at 6 weeks and 10 weeks after baseline. Participants were compensated US $50 per completed assessment.

### Study Conditions

#### Overview

After completing the baseline assessment, participants in the treatment condition were emailed a link to the MoodHacker mobile Web app and instructed to use the app for the next 6 weeks. Participants in the alternative care condition were sent an email with links to credible online resources about depression and instructed to review it on their own schedule for the next 6 weeks. Alternative care participants were offered use of the MoodHacker app upon completion of the 10-week assessment.

#### Treatment: MoodHacker App Intervention

The MoodHacker responsive mobile Web app was designed to educate users about depression and the benefits of CBT-based strategies to improve mood self-management and to activate (1) daily mood and activity monitoring, (2) increased engagement in positive behavioral activities, (3) decreased negative thinking and increased positive thinking, (4) increased practice of gratitude, mindfulness, and strength-based cognitions and behaviors, and (5) daily practice of these skills to improve depression symptoms and increase resilience to future mood disturbances.

**Figure 2:**
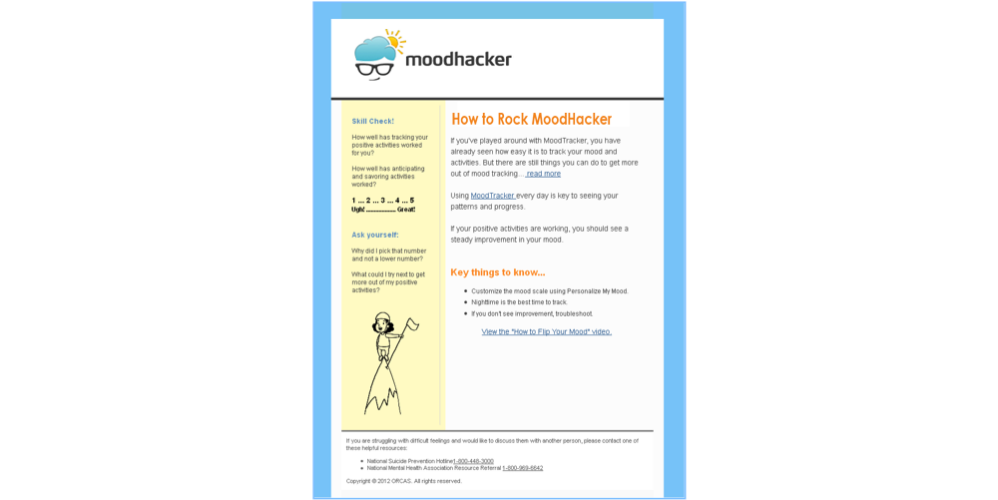
Sample MoodHacker daily email.

The 6-week MoodHacker intervention is structured around the key learning and behavioral objectives above. Content was adapted from the CWD group therapy course [21], and enhanced with mindfulness-based [33] and other evidence-based positive psychology strategies [29,31,36]. Content is sequenced to follow the enhanced CWD approach and delivered through daily emails, in-app messaging, and in the *Articles & Videos* library. Daily emails (see Figure 2) are sent to engage users in program content, provide sequenced guidance through the learning objectives in the articles and whiteboard-style videos, give tips for getting the most out of MoodHacker, and prompt the user to track their mood and activities daily. Users are encouraged to view the articles and videos as ordered, but viewing is not restricted, and users can view content according to their interests. The emails, articles, and videos promote daily use of the featured cognitive and behavioral skills outside the app experience.

**Figure 3:**
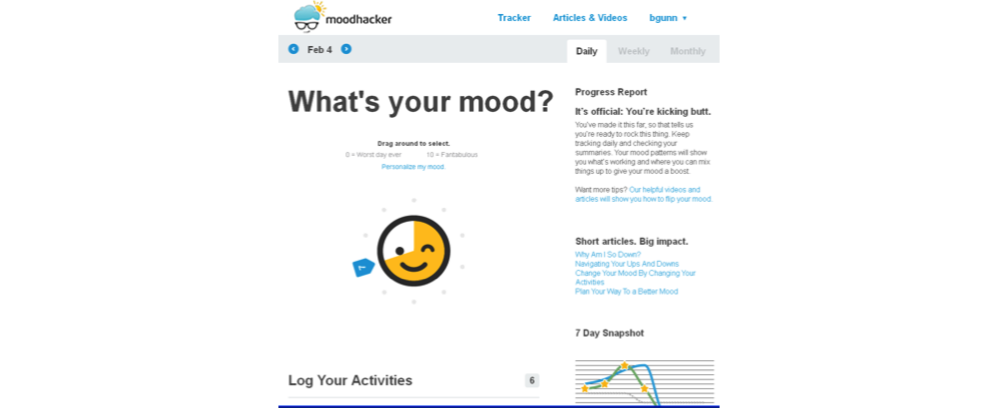
MoodHacker daily mood and activity tracking page.

**Figure 4:**
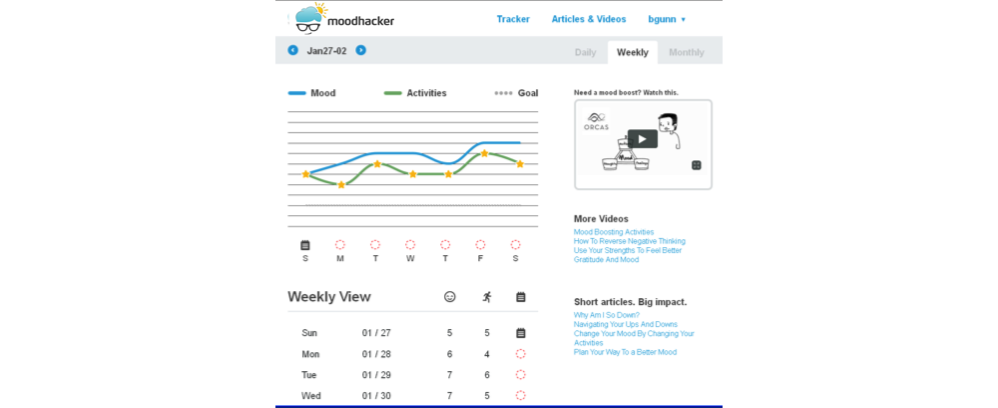
MoodHacker weekly mood and activity graph page.

Users are encouraged to monitor their mood and positive cognitive and behavioral activities daily via mobile and/or desktop access to MoodHacker. The tracker shows daily (Figure 3), weekly (Figure 4), and monthly views to highlight progress over time and patterns between positive activities and mood ratings. A customizable list of positive activities is presented by domain and promotes the types of activities known to have the highest impact (ie, social, physical, and success). The tracker includes a journaling feature for users to note mood triggers, experiences with the suggested activities, or personal information about their day. A goal-setting feature allows the user to set a goal for the number of positive activities they want to accomplish each day.

Participants in the treatment arm accessed the password-protected MoodHacker app with unique usernames and passwords provided for the study. Although daily app use was recommended in the app content, participants were not required to achieve any app use milestones to advance through the app experience. Participants received no clinical support as part of the study.

Development of the MoodHacker app was undertaken by a multidisciplinary team of researchers and developers at ORCAS; input was incorporated from experts with extensive experience in CBT-based self-management interventions for adults with depression and the benefits of positive psychology. Additional program modifications were made based on data from individual interviews and iterative user testing with the population of interest during the formative and production phases of the project. The randomized trial was conducted with the first version of the MoodHacker app. No changes were made to the app during the study period.

#### Alternative Care: Vetted Websites

Alternative care participants received an email with links to vetted online information about depression from Help Guide [72], the Mayo Clinic, Mental Health America [73], and the National Institute of Mental Health [74]; they were encouraged to browse these sites on their own schedule for 6 weeks. The educational links were emailed after the baseline assessment. Participants in the alternative care group were then given access to the MoodHacker program after the 10-week assessment.

### Outcome Measures

#### Overview

Online surveys were used to assess the following: (1) depression symptoms, (2) behavioral activation, (3) negative cognitions, (4) worksite outcomes, (5) knowledge, and (6) user satisfaction and program usability. The primary outcome measure was depression symptoms, which was the target of the intervention. Secondary or exploratory measures included the following: (1) potential mediators (ie, behavioral activation, negative cognitions, and knowledge) and (2) potential worksite outcomes that may be influenced by improvement in worker depression. Participants completed self-report assessments at each of the assessment points: baseline, 6 weeks, and 10 weeks.

#### Demographics

Demographic data were collected during the screening process, including the following: (1) gender, (2) age, (3) race/ethnicity, (4) marital status, (5) highest completed education, (6) household income, and (7) employment status.

#### Depression Symptoms

Depressive symptomatology was assessed at each assessment point using the self-reported PHQ-9 (alpha = .71) [75] to assess the nine symptoms of major depression, based on the Diagnostic and Statistical Manual of Mental Disorders, 4th edition (DSM-IV). The PHQ-9 has been shown to be a reliable and valid brief depression assessment tool [76]. Scores are summed, with higher scores indicating higher dysfunction.

#### Behavioral Activation

How actively individuals are taking care of themselves, including making positive life choices, was expected to increase as a result of the intervention. Behavioral activation was measured using the Behavioral Activation for Depression Scale (BADS) Short Form [77,78]. This scale consists of nine items (alpha = .67). Items are summed, with lower scores indicating higher dysfunction.

#### Negative Thinking

Change in negative thinking was assessed using the Automatic Thoughts Questionnaire-Revised (ATQ-R) scale Short Form [79]. A 12-item adaptation of the ATQ-R instrument asked respondents to rate how many times over the past week they have had thoughts that are consistent with 12 negative self-statements (alpha = .92). Items are summed, with higher scores indicating higher dysfunction.

#### Knowledge

Participants were assessed for increase in knowledge about depression. The scale consisted of 14 multiple-choice items developed for this study based on the 12 learning objectives addressed in the MoodHacker articles and videos. Higher scores indicate higher knowledge. Test/retest reliability over a 6-week interval for this scale was .65.

#### Worksite Outcomes

Worker productivity was assessed using the Work Limitations Questionnaire (WLQ) (alpha = .87) [80-82]. The WLQ Short Form consists of eight items divided into four subscales measuring the degree to which a person was limited in their job’s (1) time demands, (2) physical demands, (3) mental demands, and (4) output demands. Work productivity loss was calculated using methods outlined by Lerner and colleagues [83], with higher scores indicating greater loss in worker productivity.

Productivity loss due to work absence was assessed using the two-item WLQ Work Absence Module, which asks about the number of full days and part days missed in the last 2 weeks due to health problems or medical care [84]. The percentage of productivity lost due to absences is the ratio of total missed hours to total usual work hours in a 2-week time frame [85]. Higher scores indicate greater work loss due to absence from work.

Worksite outcomes were also assessed using the Workplace Outcome Suite (WOS) (alpha = .74-.88) [86]. This instrument is designed as an open-access instrument to facilitate empirical research on EAP interventions. The suite contains five scales, with five items each, that assess workplace distress, absenteeism, presenteeism, work engagement, and life satisfaction. Each scale is summed separately, with three scales—workplace distress, absenteeism, and presenteeism—indicating dysfunction, and two scales—work engagement and life satisfaction—indicating positive workplace outcomes.

#### User Satisfaction and Program Usability

At 6 weeks, treatment participants completed the System Usability Scale, which is a quantitative measure of program ease of use [87]. The scale includes 10 items, and users were asked to what degree they agreed or disagreed with program use and satisfaction statements on a 6-point scale from 1 (strongly disagree) to 6 (strongly agree). Items were scored using methods outlined by Bangor and colleagues [88], with a higher score indicating higher usability.

### Statistical Analysis

Statistical power calculations for the analysis of covariance (ANCOVA) indicated that a sample size of 300 yielded sufficient power (>.80) to detect a condition effect of Cohen’s *d*=0.34 or larger (moderately small effect size). A recent meta-analysis of Internet-based CBT interventions for depression [59] had found a mean effect size of *d*=0.32 for change in depressive symptoms and the mean effect size obtained in a randomized pilot study evaluating an abbreviated prototype of this app was *d*=1.05. Thus, this sample size provided adequate statistical power to detect the anticipated effects for the primary outcomes of interest.

Univariate effects of intervention condition, EAP access, and their interaction on outcome measures were examined using between-subjects ANCOVA, adjusting for pretest outcomes. These analyses were conducted to evaluate effects on outcome measures assessed at both 6-week and 10-week follow-up. If the condition by EAP access interaction was significant for an outcome measure, separate subpopulation ANCOVA analyses were conducted on that outcome measure for subjects with and without EAP access. We explored dose-response relationships and self-monitoring participation within the treatment group by correlating process indicants with change in outcome measures. All subjects were included in intent-to-treat (ITT) analyses at each follow-up. Prior to conducting these analyses, we employed the single imputation procedure available in SPSS, version 21.0 (IBM Corp) to account for missing data. Alpha was set to *P*<.05, two-tailed, for all tests.

## Results

### Baseline Equivalency and Attrition

The expectation of baseline equivalency due to random assignment of groups was examined. The treatment and alternative care groups were compared on demographic characteristics and outcome measures collected at pretest. Contingency table analyses and *t* tests were conducted on categorical and continuous measures, respectively. The groups did not significantly differ on any demographic characteristics or pretest outcome measures. See Table 1 for demographic descriptive data.

**Table 1:** Demographic characteristics by condition.

Demographic characteristics		Treatment (n=150)	Alternative care (n=150)
Age in years, mean (SD)		40.6 (11.5)	40.7 (11.2)
Number of children, mean (SD)		2.0 (1.2)	2.0 (1.2)
**Ethnicity, n (%)**			
	Hispanic/Latino	22 (14.6)	21 (14.0)
	Non-Hispanic/Latino	128 (85.3)	127 (84.7)
**Race, n (%)**			
	Asian	3 (2.0)	6 (4.0)
	Hawaiian	1 (0.7)	0 (0)
	African American	32 (21.3)	25 (16.7)
	Caucasian	102 (68.0)	105 (70.0)
	Mixed	9 (6.0)	9 (6.0)
**Gender, n (%)**			
	Female	112 (74.6)	118 (78.7)
	Male	37 (24.7)	32 (21.3)
**Employment status, n (%)**			
	Full time	84 (56.0)	92 (61.3)
	Part time	53 (35.3)	46 (30.7)
	Self-employed	13 (8.7)	12 (8.0)
**Marital status, n (%)**			
	Married/living with partner	78 (52.0)	72 (48.0)
	Divorced	22 (14.7)	23 (15.3)
	Widowed	3 (2.0)	2 (1.3)
	Separated	5 (3.3)	5 (3.3)
	Single	42 (28.0)	47 (31.3)
**Education, n (%)**			
	High school diploma or GED^a^	9 (6.0)	20 (13.3)
	Some college, associates, trade school, military	54 (36.0)	47 (31.3)
	College degree (ie, BA^b^ or BS^c^)	60 (40.0)	54 (36.0)
	Graduate school/professional training	27 (18.0)	28 (18.7)
**Annual household income (US $), n (%)**			
	$19,999 or less	21 (14.0)	22 (14.6)
	$20,000-$39,999	46 (30.7)	42 (28.0)
	$40,000-$59,999	25 (16.7)	29 (19.3)
	$60,000-$79,999	30 (20.0)	29 (19.3)
	$80,000-$99,999	12 (8.0)	14 (9.3)
	$100,000 or more	16 (10.7)	13 (8.7)
**EAP** ^d^ **access, n (%)**			
	Yes	46 (30.6)	45 (30.0)
	No	99 (66.0)	100 (66.7)

^a^GED: General Educational Development.

^b^BA: Bachelor of Arts.

^c^BS: Bachelor of Science.

^d^EAP: employee assistance program.

The extent to which attrition threatened the internal and external validity of the study was evaluated using contingency table analyses and analysis of variance (ANOVA). Participants who completed each of the follow-up assessments were compared to those who did not complete that follow-up with respect to demographic characteristics and pretest outcome measures. We also conducted analyses to test whether outcome variables were differentially affected across conditions by attrition. These latter analyses examined the effects of condition, attrition status, and their interaction on pretest outcomes. Examination of attrition between pretest and 6-week follow-up revealed only 10 out of 150 (6.7%) treatment participants did not complete the assessment compared to 4 out of 150 (2.7%) alternative care participants. Only 10 out of 150 (6.7%) treatment participants did not complete the 10-week follow-up assessment compared to 5 out of 150 (3.3%) alternative care participants. Attrition rates did not significantly differ by condition. Moreover, we found no statistically significant differences in demographic characteristics or baseline outcomes by attrition status, nor did we find any statistically significant interactions between attrition and condition predicting baseline outcomes, suggesting that attrition was not systematic.

Analyses compared baseline demographic data of subjects with EAP access versus those without EAP access. Subjects with EAP access had significantly more children (*P*=.003), consisted of fewer Hispanics (*P*=.01), were more likely to have full-time employment (*P*=.001), had a higher level of education (*P*=.047), and had a greater income (*P*=.001).

### Intervention Effects

Table 2 provides ANCOVA results for all outcome measures at 6-week and 10-week follow-up. Multimedia Appendix 2 provides means and standard deviations for each outcome by assessment time and condition, along with pretest to 6-week follow-up and pretest to 10-week follow-up outcome analyses.

#### Primary Outcome: Depression Symptoms

From pretest to 6-week follow-up, the ANCOVA with the full sample found statistically significant program effects on depression symptoms (PHQ-9) (*P*=.01, partial eta^2^ = .021). However, a statistically significant condition-by-user-EAP-access interaction effect was also obtained (*P*=.05, partial eta^2^ = .013), indicating differential program effects depending upon subjects’ access to an EAP. Separate subpopulation analyses indicated significant positive program effects for subjects with EAP access (*P*=.004, partial eta^2^ = .093) and no program effects for subjects without EAP access (*P*=.66, partial eta^2^ = .001). From pretest to 10-week follow-up, the condition-by-EAP-access interaction effect was not statistically significant (*P*=.21, partial eta^2^ = .005), so subpopulation analyses were not indicated. The ANCOVA with the full sample found that there were no program effects (*P*=.17, partial eta^2^ = .006) at 10-week follow-up.

#### Mediator Outcomes: Behavioral Activation, Negative Thoughts, and Knowledge

From pretest to 6-week follow-up, the ANCOVA with the full sample found statistically significant positive effects for the program on each mediator measure: BADS (*P*=.004, partial eta^2^ = .027), ATQ-R (*P*=.01, partial eta^2^ = .020), and knowledge (*P*=.02, partial eta^2^ = .017). The condition-by-EAP-access interaction effects were not statistically significant on any of the mediator measures, indicating that there was no need for subpopulation analyses. From pretest to 10-week follow-up, the condition-by-EAP-access interaction effects were not statistically significant on any of the mediator measures. The ANCOVA with the full sample found statistically significant program effects on BADS (*P*=.01, partial eta^2^ = .021), but not on ATQ-R (*P*=.34, partial eta^2^ = .003) or knowledge (*P*=.55, partial eta^2^ = .001).

#### Worksite Outcomes

From pretest to 6-week follow-up, the condition-by-EAP-access interaction effects were statistically significant on the WLQ productivity loss measure (*P*=.048, partial eta^2^ = .016), the WLQ work absence measure (*P*=.048, partial eta^2^ = .016), and the workplace distress measure (*P*=.03, partial eta^2^ = .017). Consequently, subpopulation analyses were carried out for these measures only. The ANCOVA with the full sample at 6-week follow-up found statistically significant program effects on WLQ work absence (*P*=.003, partial eta^2^ = .032). No statistically significant program effects were found on the following: WLQ productivity loss (*P*=.20, partial eta^2^ = .007), WOS absenteeism (*P*=.16, partial eta^2^ = .007), WOS presenteeism (*P*=.09, partial eta^2^ = .010), WOS engagement (*P*=.75, partial eta^2^ = .001), or WOS life satisfaction (*P*=.12, partial eta^2^ = .008). However, separate subpopulation analyses at 6-week follow-up on the WLQ productivity loss measure indicated significant positive effects of the program for subjects with EAP access (*P*=.047, partial eta^2^ = .052) and no program effects for subjects without EAP access (*P*=.60, partial eta^2^ = .002). Similarly, subpopulation analyses at 6-week follow-up on the WLQ work absence measure indicated significant positive effects of the program for subjects with EAP access (*P*=.02, partial eta^2^ = .070) and no program effects for subjects without (*P*=.51, partial eta^2^ = .002). Lastly, the WOS workplace distress measure indicated significant positive effects of the program for subjects with EAP access (*P*=.007, partial eta^2^ = .080) and no program effects for subjects without (*P*=.64, partial eta^2^ = .001).

From pretest to 10-week follow-up, the condition-by-EAP-access interaction effects were statistically significant on the WLQ work absence measure (*P*=.04, partial eta^2^ = .016). Consequently, subpopulation analyses were carried out for this measure only. The ANCOVA with the full sample at 10-week follow-up found statistically significant program effects only on the WLQ work absence measure (*P*=.02, partial eta^2^ = .022). Separate subpopulation analyses at 10-week follow-up on the WLQ work absence measure indicated significant positive effects of the program for subjects with EAP access (*P*=.03, partial eta^2^ = .060) and no program effects for subjects without EAP access (*P*=.78, partial eta^2^ = 0).

#### Program Utilization, Satisfaction, and Usability

On average, participants in the treatment arm logged into the MoodHacker app 16.0 times (SD 13.3, range 1-49) for a total duration of 1.3 hours (SD 1.3, range 0-6.5) between pretest and 6-week follow-up. The average rating of program satisfaction was 4.6 (SD 1.0) on a 6-point scale, indicating that the participants were mostly satisfied with the intervention. Participants also completed the System Usability Scale [87] at the 6-week follow-up, which provides a quantitative measure of program ease of use. The average System Usability Scale score was 79.7 (SD 17.1), corresponding to a usability grade of B+ for the intervention program.

**Table 2:** ANCOVA^a,b^ results for all outcome measures at 6-week and 10-week follow-up.

Outcome measure/condition			Pretest to 6-week follow-up condition effect			Pretest to 10-week follow-up condition effect		
			*F*	*P*	Partial eta^2^	*F*	*P*	Partial eta^2^
**Depression symptoms, PHQ-9** ^**c,d**^								
	All subjects (n=300)		6.20	*.01* ^e^	*.021*	0.93	.34	.003
	EAP^f^ (n=91)		9.00	*.004*	*.093*	N/A^g^	N/A	N/A
	Non-EAP (n=209)		0.20	.66	.001	N/A	N/A	N/A
**Mediator outcomes**								
	BADS^h,i^ (n=300)		8.26	*.004*	*.027*	6.39	*.01*	*.021*
	ATQ-R^d,j^ (n=300)		6.09	*.01*	*.020*	1.90	.17	.006
	Knowledge^i^ (n=300)		5.15	*.02*	*.017*	0.37	.55	.001
**Worksite outcomes**								
	**WLQ** ^k^ **productivity loss** ^d^							
		All subjects (n=300)	1.66	.20	.007	1.02	.31	.004
		EAP (n=91)	*4.09*	*.047*	*.052*	2.14	.15	.027
		Non-EAP (n=209)	0.28	.60	.002	0.10	.76	.001
	**WLQ work absence** ^d^							
		All subjects (n=300)	*8.69*	*.003*	*.032*	*5.92*	*.02*	*.022*
		EAP (n=91)	*6.13*	*.02*	*.070*	*5.19*	*.03*	*.060*
		Non-EAP (n=209)	0.44	.51	.002	0.08	.78	0
	**WOS** ^l^ **workplace distress** ^d^							
		All subjects (n=300)	N/A	N/A	N/A	1.32	.25	.004
		EAP (n=91)	7.63	*.007*	*.080*	N/A	N/A	N/A
		Non-EAP (n=209)	0.22	.64	.001	N/A	N/A	N/A
	WOS absenteeism^d^ (n=300)		1.97	.16	.007	1.24	.27	.004
	WOS presenteeism^d^ (n=300)		2.92	.09	.010	1.40	.24	.005
	WOS engagement^i^ (n=300)		0.10	.75	.001	0.01	.99	.001
	WOS life satisfaction^i^ (n=300)		2.50	.12	.008	0.65	.42	.002

^a^ANCOVA: analysis of covariance.

^b^Intent-to-treat model: missing data were addressed via a single imputation procedure.

^c^PHQ-9: Patient Health Questionnaire-9.

^d^A higher score represents more dysfunction.

^e^Values in italics represent significant results, *P*<.05.

^f^EAP: employee assistance program.

^g^N/A: not applicable.

^h^BADS: Behavioral Activation for Depression Scale.

^i^A lower score represents more dysfunction.

^j^ATQ-R: Automatic Thoughts Questionnaire-Revised.

^k^WLQ: Work Limitations Questionnaire.

^l^WOS: Workplace Outcome Suite.

## Discussion

### Principal Findings

This randomized effectiveness trial examined the effect of the MoodHacker mobile Web app on depression symptoms, important cognitive and behavioral mediators, and workplace outcomes among employed adults with and without access to EAP services. The MoodHacker app produced significant effects from pretest to 6-week follow-up for all subjects compared to alternative care subjects on (1) the clinical outcome measure (ie, depression symptoms), with much stronger results among those with EAP access, (2) all three mediating outcome measures (ie, behavioral activation, negative thoughts, and depression knowledge), and (3) one of the worksite outcome measures (ie, work absence). Among subjects with EAP access, larger effects were found on depression symptoms, productivity loss, work absence, and workplace distress. Significant effects were maintained at 10-week follow-up for work absence for all subjects, with much stronger results among those with EAP access. The effect sizes on depression symptoms in this study (partial eta^2^ = .021 for the full population and partial eta^2^ = .093 for those with EAP access, which convert to approximately Cohen’s *d*=0.30 and 0.64, respectively) are comparable to those reported for previous meta-analyses of self-guided, Internet-based CBT programs (Cohen’s *d*=0.21-0.31) [42,44].

These findings suggest that the approach used to activate CBT-based skills in MoodHacker was effective and begin to build an evidence base for light-touch, CBT-based mobile apps for depression self-management. Because program effects were small-to-medium size, modifications to increase the potency of the intervention are warranted. Evidence from prior online CBT-based programs suggests that supporting the mobile intervention with counselors, such as those working in EAPs, is likely to increase both adherence to the app and efficacy in reducing depression symptoms [42,43,47,89].

At the 10-week assessment, only effects on work absence remained significant. Effects on depression symptoms were no longer significant, possibly because the daily emails ended after 6 weeks; the emails included prompts to view content, introduction of key concepts and skills, tips to optimize use of the program, and encouragement to track mood and activities. Although our results are consistent with a recent meta-analysis of computerized CBT programs showing that results of computer-based CBT programs typically attenuate over time [45], evidence from other studies suggests that app adherence and, thus, efficacy might be improved by extending program prompts and psychoeducational messaging beyond the original 6-week intervention period and/or providing mobile-friendly prompts (eg, text messages and app notifications) in addition to the emails utilized here [65,90]. More research is needed to determine the optimal level and type of program contact that is needed to retain program efficacy.

### Employee Assistance Program Participants Versus Non-Employee Assistance Program Participants

The inclusion of subjects without access to EAP services provided an important opportunity to evaluate program efficacy with this population and to compare program efficacy for subjects with and without EAP access. It was expected that the subjects with access to EAP services who chose to participate in this study were likely to be quite similar to real-world individuals who might elect to use a mobile or online program in conjunction with those services. Thus, these subjects provided a “real-world” effectiveness trial. The effect sizes found on depression symptoms, work absence, and productivity loss among the targeted EAP population are quite encouraging and suggest that EAPs offering use of the MoodHacker app may reasonably expect to find significant depression-related improvements in their employee populations. Conclusions regarding program effects for non-EAP individuals are less clear.

In many cases, the subjects without EAP access were recruited without the endorsement of a trusted partner, most notably when recruitment was through entities such as eHow, Chambers of Commerce, eHealth websites, and especially Craigslist. This raises questions regarding the motivation of these subjects for participation. For example, participants who presented to their EAP with depression symptoms and were willing to use a behavior-change program might reasonably be expected to be motivated to change their depression. In contrast, participants recruited from the other entities listed above might have been more motivated by the financial compensation offered, rather than a desire to improve their depression symptoms. Further evidence of skewed motivations in this recruitment group is indicated in the very high numbers of individuals who were screened out by reporting much higher than expected rates of mania, and to a lesser extent, depressive symptoms during the online screening.

Analyses of baseline data indicated that the non-EAP subjects had lower incomes, were less likely to be fully employed, and had less education. Since these three factors are also related to poorer outcomes in depression, these factors may at least partially explain why the program was not as effective for individuals with no EAP access [91,92]. While the veracity of such attributions regarding motivation for participation is difficult to ascertain, the lack of program effects on depression symptoms in the non-EAP participants is consistent with this notion.

### Study Limitations

We acknowledge several limitations in this initial efficacy trial and offer some caution in interpreting the findings. First, although random assignment was used, all the participants volunteered for the study and thus represent a convenience sample of interested individuals and cannot be considered representative of the general population. Second, participants completed self-report surveys, the validity and reliability of which may be somewhat suspect. Third, the reliability of some measures is only moderate and this may have attenuated the effect size of the intervention effects found in the study. Fourth, while the attrition rates in the study were relatively low, subjects were compensated for completing assessments. Thus, it cannot be concluded that the subject completion rate found here would occur at the same rate without compensation for participation. Lastly, attenuation of outcomes at 10-week follow-up suggests a need for more potent activation of CBT-based skills or a need for extended app contacts to drive continued engagement. Future research will explore the frequency and type of app contacts to optimize extended engagement and improve both short- and long-term efficacy of this light-touch, mobile approach to activating use of CBT skills to self-manage depressive symptomatology.

### Summary

Given the high prevalence of depression and the fact that most adults with depression never receive treatment, there is a critical need for effective interventions that can be widely and cost-effectively disseminated through multiple delivery channels. The MoodHacker mobile Web app has demonstrated potential for dissemination (1) as a self-guided intervention for individuals unwilling or unable to seek in-person depression services and (2) through EAP and similar health and wellness organizations supporting employed adults and their families. Ideally, exposure to MoodHacker needs to be extended beyond the 6-week time frame to maintain user engagement and improve longer-term efficacy. Further, the implementation and integration of MoodHacker within EAPs and similar organizations that can provide guidance from counselors seems likely to augment the effectiveness of the MoodHacker app.

## Appendix

Multimedia Appendix 1 CONSORT-EHEALTH checklist V1.6.1 [71].

Multimedia Appendix 2 Pretest, 6-week follow-up, and 10-week follow-up descriptive statistics and ANCOVA results for all outcome measures.

## Abbreviations

ANCOVA: analysis of covariance
ANOVA: analysis of variance
ATQ-R: Automatic Thoughts Questionnaire-Revised
BA: Bachelor of Arts
BADS: Behavioral Activation for Depression Scale
BS: Bachelor of Science
CBT: cognitive behavioral therapy
CWD: Coping with Depression
DSM-IV: Diagnostic and Statistical Manual of Mental Disorders, 4th edition
EAP: employee assistance program
EMA: ecological momentary assessment
GED: General Educational Development
IRB: Institutional Review Board
ITT: intent to treat
N/A: not applicable
PHQ-2: Patient Health Questionnaire-2
PHQ-9: Patient Health Questionnaire-9
SMS: short message service
WLQ: Work Limitations Questionnaire
WOS: Workplace Outcome Suite
