# Supplementary material for: MoodHacker Mobile Web App With Email for Adults to Self-Manage Mild-to-Moderate Depression: Randomized Controlled Trial
Source: JMIR Mhealth Uhealth. 2016 Jan 26;4(1):e8. doi: 10.2196/mhealth.4231 (PMC4748138; doi:10.2196/mhealth.4231)
Supplement: Multimedia Appendix 2 [file mhealth_v4i1e8_app2.pdf]

Multimedia Appendix 2. Pre, 6-Week Follow-up, and 10-Week Follow-up Descriptive Statistics and ANCOVA Results for All Outcome Measures

Outcome Measure/ Condition	Pretest	6-Week Follow-up		10- Week Follow-up		Pre to 6-Week Follow-up Condition Effect			Pre to 10-Week Follow-up Condition Effect		
	M (*SD*)	*M* (*SD*)	*Adj M*	*M* (*SD*)	*Adj M*	*F*-test	*p*-value	(Partial Eta^2)	*F*-test	*p*-value	(Partial Eta^2)
**Depression Symptoms: PHQ[1]**											
All Subjects (N=300)						6.20	**.013**	**(.021)**	0.93	.336	(.003)
Treatment	13.2 (4.3)	-	-	8.8 (5.1)	8.9						
Alternative Care	13.6 (3.8)	-	-	9.5 (5.0)	9.4						
EAP (N=91)						9.00	**.004**	(**.093**)	-	-	-
Treatment	12.6 (3.2)	7.7 (4.5)	7.8	-	-						
Alternative Care	13.2 (3.4)	10.5 (4.0)	10.4	-	-						
Non-EAP (N=209)						0.2	.655	(.001)	-	-	-
Treatment	13.5 (4.7)	9.7 (5.4)	9.8	-	-						
Alternative Care	13.7 (3.9)	10.2 (4.8)	10.1	-	-						
**Mediator Outcomes**											
BADS[2] (N=300)						8.26	**.004**	(**.027**)	6.39	**.012**	(**.021**)
Treatment	21.3 (7.4)	27.1 (10.0)	27.2	27.5 (10.1)	27.7						
Alternative Care	22.0 (6.9)	24.9 (8.1)	24.7	25.7 (9.0)	25.6						
ATQ-R[1] (N=300)						6.09	**.014**	(**.020**)	1.90	.169	(.006)
Treatment	35.5 (11.2)	28.1 (11.1)	28.3	27.0 (11.0)	27.2						
Alternative Care	36.2 (10.4)	30.4 (10.5)	30.2	28.8 (11.9)	28.6						
Knowledge[2] (N=300)						5.15	**.024**	(**.017**)	0.37	.545	(.001)
Treatment	57.0 (18.3)	63.4 (19.7)	64.5	63.3 (18.9)	64.2						
Alternative Care	60.0 (15.5)	60.5 (18.5)	59.4	63.0 (16.6)	62.0						

**Worksite Outcomes**											
WLQ Productivity Loss[1]											
All Subjects (N=300)						1.66	.199	(.007)	1.02	.314	(.004)
Treatment	6.0 (7.3)	9.0 (5.5)	8.9	8.6 (5.3)	8.5						
Alternative Care	4.6 (6.5)	9.5 (4.7)	9.5	9.0 (5.5)	9.1						
EAP (N=91)						**4.09**	**.047**	(**.052**)	2.14	.147	(.027)
Treatment	4.0 (6.5)	7.1 (4.6)	7.1	4.2 (6.6)	6.8						
Alternative Care	3.7 (6.2)	9.3 (5.0)	9.3	3.7 (6.3)	8.5						
Non-EAP (N=209)						0.28	.595	(.002)	0.10	.755	(.001)
Treatment	7.0 (7.6)	10.2 (5.5)	10.1	9.7 (5.2)	9.6						
Alternative Care	5.2 (6.7)	9.6 (4.7)	9.7	9.2 (5.5)	9.3						
WLQ Work Absence[1]											
All Subjects (N=300)						**8.69**	**.003**	**(.032)**	**5.92**	**.016**	**(.022)**
Treatment	0.35 (0.25)	0.28 (0.16)	0.27	0.23 (0.16)	0.22						
Alternative Care	0.30 (0.18)	0.29 (0.21)	0.31	0.24 (0.16)	0.25						
EAP (N=91)						**6.13**	**.015**	(**.070**)	**5.19**	**.025**	**(.060)**
Treatment	0.33 (0.21)	0.26 (0.14)	0.25	0.23 (0.16)	0.22						
Alternative Care	0.31 (0.18)	0.35 (0.27)	0.35	0.30 (0.21)	0.30						
Non-EAP (N=209)						0.44	.507	(.002)	0.08	.775	(.000)
Treatment	0.37 (0.27)	0.29 (0.18)	0.28	0.24 (0.16)	0.22						
Alternative Care	0.30 (0.19)	0.27 (0.17)	0.29	0.21 (0.23)	0.23						
WOS Workplace Distress[1]											
All Subjects (N=300)						–	–	–	1.32	.252	(.004)
Treatment	16.1 (4.8)	–	–	14.3 (5.2)	14.1						
Alternative Care	15.3 (5.0)	–	–	14.2 (5.3)	14.5						
EAP (N=91)						7.63	**.007**	(**.080**)	–	–	–
Treatment	15.6 (4.0)	12.8 (4.9)	12.4	–	–						

Alternative Care	14.6 (4.5)	14.2 (4.7)	14.6	–	–						
Non-EAP (N=209)						0.22	.642	(.001)	–	–	–
Treatment	16.3 (5.1)	15.0 (5.5)	14.8	–	–						
Alternative Care	15.7 (5.2)	14.3 (5.6)	14.5	–	–						
WOS Absenteeism[1] (N=300)						1.97	.161	(.007)	1.24	.267	(.004)
Treatment	39.7 (56.8)	22.7 (39.1)	21.2	21.7 (40.0)	20.4						
Alternative Care	30.9 (38.2)	23.5 (36.8)	24.9	21.9 (40.3)	23.2						
WOS Presenteeism[1] (N=300)						2.92	.089	(.010)	1.40	.238	(.005)
Treatment	18.3 (4.3)	14.6 (5.6)	14.6	14.4 (5.6)	14.3						
Alternative Care	18.2 (4.9)	15.5 (5.5)	15.5	15.2 (5.8)	15.2						
WOS Engagement[2] (N=300)						0.10	.748	(.001)	0.01	.993	(.001)
Treatment	13.7 (4.7)	14.5 (4.6)	14.9	14.4 (4.6)	14.8						
Alternative Care	14.8 (4.2)	15.4 (4.7)	15.0	15.2 (4.5)	14.8						
WOS Life Satisfaction[2] (N=300)						2.50	.115	(.008)	0.65	.419	(.002)
Treatment	9.9 (3.5)	11.7 (4.0)	11.7	12.3 (1.9)	12.3						
Alternative Care	9.9 (3.6)	11.1 (4.1)	11.0	12.4 (2.2)	12.4						

Notes

M = Mean, *SD* = Standard Deviation, *Adj* = Adjusted.

[1] A higher score represents more dysfunction.

[2] A lower score represents more dysfunction.

Intent–to-treat model: Missing data were addressed via a single imputation procedure.
